# Analysis of statistical and standard algorithms for detecting muscle onset with surface electromyography

**DOI:** 10.1371/journal.pone.0177312

**Published:** 2017-05-10

**Authors:** Matthew S. Tenan, Andrew J. Tweedell, Courtney A. Haynes

**Affiliations:** United States Army Research Laboratory, Human Research and Engineering Directorate, Integrated Capability Enhancement Branch, Aberdeen Proving Ground, MD, United States of America; Tianjin University, CHINA

## Abstract

The timing of muscle activity is a commonly applied analytic method to understand how the nervous system controls movement. This study systematically evaluates six classes of standard and statistical algorithms to determine muscle onset in both experimental surface electromyography (EMG) and simulated EMG with a known onset time. Eighteen participants had EMG collected from the biceps brachii and vastus lateralis while performing a biceps curl or knee extension, respectively. Three established methods and three statistical methods for EMG onset were evaluated. Linear envelope, Teager-Kaiser energy operator + linear envelope and sample entropy were the established methods evaluated while general time series mean/variance, sequential and batch processing of parametric and nonparametric tools, and Bayesian changepoint analysis were the statistical techniques used. Visual EMG onset (experimental data) and objective EMG onset (simulated data) were compared with algorithmic EMG onset via root mean square error and linear regression models for stepwise elimination of inferior algorithms. The top algorithms for both data types were analyzed for their mean agreement with the gold standard onset and evaluation of 95% confidence intervals. The top algorithms were all Bayesian changepoint analysis iterations where the parameter of the prior (*p*_0_) was zero. The best performing Bayesian algorithms were *p*_0_ = 0 and a posterior probability for onset determination at 60–90%. While existing algorithms performed reasonably, the Bayesian changepoint analysis methodology provides greater reliability and accuracy when determining the singular onset of EMG activity in a time series. Further research is needed to determine if this class of algorithms perform equally well when the time series has multiple bursts of muscle activity.

## Introduction

In biomechanics, the off-line analysis of electromyography (EMG) is used to add a physiologic context to observed patterns of movement [1] or specific events during movement, such as heel-strike in walking [2]. During a defined movement, the EMG from two different muscles may also be compared [3] if theory dictates that differential activation may cause or be a predisposing factor towards injury. Generally, there are three parameters of interest: EMG amplitude, EMG frequency content, and EMG timing. Each of these measures may be influenced by the determination of signal onset, depending on the methodologies employed.

As EMG signals are prone to noise, the precise identification of signal onset is difficult and the earliest methods for detecting EMG onset involved visual inspection of EMG signals. With this technique, determination of EMG onset is somewhat subjective and based on individual differences in the perception of the signal. This difficulty is increased when the underlying EMG signal is noisy and the contraction amplitude is low. Computational methods remove the subjectivity and investigator variance from the analysis process. Many of the computational methods used are iterations of David Winter’s suggestions in his ubiquitous book Biomechanics and Motor Control of Human Movement [4]. These methods commonly include full-wave rectification of the signal, application of a low-pass filter, and use of a sliding window to determine when the amplitude of the signal exceeds some predetermined threshold, typically 1–3 standard deviations over a baseline amplitude. Within this methodology, different investigators select different low-pass filters, different window lengths for the sliding window, and different amplitude thresholds. It has been demonstrated that preconditioning the EMG signal with the Teager-Kaiser energy operator (TKEO) improves onset detection when applied to variations of Winter’s methodology [5, 6]. A method using sample entropy (SampEn) within a sliding window has also demonstrated results similar the Teager-Kaiser energy method, but is more robust to spurious artifact data [7]. The wide-array of EMG onset algorithms employed suggest that none of the currently used algorithms are sufficient for the breadth of ways in which EMG is implemented in biomechanics. The new methodologies, TKEO and SampEn, derive from the research areas of acoustics [8, 9] and electrocardiography [10, 11], respectively. Therefore, it is reasonable to extend the search for EMG onset algorithms to those typically employed in other signal processing arenas.

The computational onset methods are commonly validated against visual detection of surface EMG [5, 12, 13]. In addition to being time consuming, visual detection has variability inherent to both natural human error as well as error between researchers. Using simulated, or modeled, surface EMG data, it is possible to create a known EMG onset; however, models are not designed to be the “same” as reality, but instead a computational aide to understanding reality [14, 15]. Furthermore, the modeling of surface EMG has been controversial [16–18] and may lead to erroneous conclusions when compared to experimental data [19]. Therefore, this study examines surface EMG onsets determined in a randomized double-blind visual method as well as a simulated surface EMG signal which has been modified to have a known onset. This two-pronged approach should assuage concerns both about the reliability of visual detection as well as the use of modulated or simulated data to represent empirically collected surface EMG.

The purpose of the present investigation is to systematically employ and validate a variety of established algorithms and econometrics-based statistical algorithms for the determination of EMG onset using experimental surface EMG data and experimental data which has been modified to have a known onset (simulated EMG). The development of an algorithm or set of algorithms that are consistently reliable for EMG onset determination should enable more objective EMG onset detection across studies, providing a standard analytic method for clinical research studies.

## Materials and methods

### Participants and ethical approval

Eighteen participants, 13 male and 5 female (33.3 ± 9.2 years), participated in the study. The inclusion criteria for participation was the absence of neurologic, cardiovascular or metabolic disorders. Furthermore, they had no previous surgeries, current or recurring pain, or injury on their dominant side. The study protocol was approved by the U.S. Army Research Laboratory Institutional Review Board and all participants gave their written informed consent in accordance with the Helsinki Declaration.

### Experimental protocol

Participants performed two independent and discrete movements as a part of the research study: knee extension and elbow flexion of the dominant limb. The dominant limb was ascertained by asking the participant which limb they would use to kick or throw a ball. In the present study, all participants were right-side dominant. For the knee extension movement, participants were seated in a stationary chair with their foot at least 10 cm from the ground and a mass of 2.3 kg was attached to their ankle. A surface EMG electrode (B&L Engineering, Tustin, CA; Ag/AgCl, circular 10mm diameter, interelectrode distance 35mm, Gain 330x, >100MΩ, CMMR 95db, 10 Hz-3.13 kHz bandwidth) was attached to the vastus lateralis approximately 2/3 the distance between the anterior superior iliac spine and the lateral side of the patella. Manual muscle tests for simple hip flexion and leg abduction were performed to limit cross-talk. For the elbow flexion movement, participants were seated in a chair with their elbow supported in 90 degrees of flexion and a 2.3 kg mass attached to their wrist. A surface EMG electrode was attached midway between the lateral epicondyle and the acromion on the the short head of the biceps brachii as determined via palpation. Prior to the placement of the EMG electrode for both movements, the skin was lightly abraded and cleaned with a 70% isopropyl alcohol solution. A 5 mm diameter adhesive pre-gelled Ag/AgCl surface electrode was placed on the subject’s ipsilateral patella as a ground. All EMG was pre-amplified with a 330x gain and A/D converted at 2048 Hz. After placement of the EMG electrodes, the process for both movements was similar. The participant was asked to perform three repetitions of the specific movement at a self-selected pace separated by at least 60 seconds of rest. No attempt was made to standardize movement speed or EMG signal-to-noise ratio as the goal of the present analysis is to determine an algorithm or class of algorithms which are valid for use in a variety of conditions. Five trials were lost due to equipment malfunction, rendering 103 trials of experimental EMG data for analysis. Since this same data was used for the creation of the simulated EMG, this resulted in a net total of 206 time series’ of data for analysis.

### Visual electromyography onset detection

A custom computer program was written in the R programming language which enabled all three researchers to visually determine and record EMG onset in a randomized and double-blind fashion. Within each researcher, the visual determinations were separated by at least 24 hours and the inter-determination period was never longer than 7 days. Prior to researcher evaluation of the EMG, the signal was bandpass filtered (10–1,000 Hz) in line with previous research to remove any substantial signal artifact [13]. The reviewing researchers were instructed to evaluate the time series for what they perceived to be the onset of muscle activity. Each researcher evaluated every trial twice and was blinded to both the study identifier as well as the movement performed. The reliability between and within investigators was high (ICC: 0.88 and 0.92, respectively); therefore, the mean of the six visual detections (two from each researcher) was used as the ‘gold standard’ from which to evaluate the algorithms [5, 13].

### Creation of simulated EMG

The experimental data collected was altered to obtain a known onset of EMG signal. A half-second increment of data (1024 samples) was extracted from the obtained signal in a trial prior to muscle contraction. During this period the muscle was always quiescent. Since the greatest singular investigator visual onset deviation from visual ‘gold standard’ was 0.32 seconds, active EMG was obtained 0.5 seconds after the visual ‘gold standard’ determination. The length of active EMG was 1 second (2048 samples). Thus, the length of all simulated trials was 1.5 seconds (3072 samples) with an objective EMG onset at 0.5 seconds. While the onset in simulated EMG is objective, this method renders a data series with an artificially profound onset since it does not contain the gradual orderly recruitment of different motor units. However, the inability to sufficiently determine an onset in the simulated EMG indicates that an algorithm is inadequate even when a clear EMG signal change occurs.

### Algorithmic electromyography onset detection

A net total of 605 algorithm iterations were tested (Table 1 and Table 2). Prior to analysis by any algorithm, the raw EMG waveform was bandpass filtered (10–1,000 Hz) to remove signal artifact [13]. Three classes of standard algorithms were examined (Table 1), including sixty-four iterations of the commonly applied linear envelope methodology [4]. The linear envelope was also tested with the Teager-Kaiser Energy Operator (TKEO) pre-processing step since this has been shown to increase accuracy [5, 6]. The Sample Entropy (SampEn) algorithm has also been shown to have accuracy similar to TKEO while being more robust to artifact [7]. In addition to testing these 129 standard EMG onset algorithms, 476 statistical algorithms for time series analysis were applied for use in EMG onset detection (Table 2).

**Table 1:** Standard EMG onset detection methodologies examined and the iterative settings used for each methodology.

**Method**	**Rectification**	**Low Pass Filtering** **or Windowing Parameters**	**Onset Threshold**	**Number of Algorithms**	**Notes**
**Linear Envelope**	Yes	2–20 Hz (incremented every 2 Hz), 25–50 Hz (incremented every 5 Hz) cut off frequency	1, 2, 3 and 15 SD of time series	64	
**Teager-Kaiser Energy Operator**	Yes	2–20 Hz (incremented every 2 Hz), 25–50 Hz (incremented every 5 Hz) cut off frequency	1, 2, 3 and 15 SD of time series	64	
**Sample Entropy**	No	32 ms windows, incremented every 4 ms	0.6	1	Zhang & Zhou 2012

All of the statistical algorithms were tested with both raw EMG and after applying a full-wave rectification pre-processing step. The full-wave rectification theoretically assists in the detection in waveform changepoints when the algorithm is based on detecting changes in the mean of the signal.

**Table 2:** Statistical EMG onset detection methodologies examined and the iterative settings used for each methodology.

**Method**	**Rectification**	**Parameters or** **Models Used**	**Onset** **Threshold**	**Number of Algorithms**	**Notes**
**General Time Series Mean/Variance**	Both	Changes in mean, variance or both	N/A	6	Default algorithm settings used unless otherwise noted
**Sequential Changepoint Detection with Parametric Methods**	Both	Models: Student, Bartlett, Generalized Likelihood Ratio, Generalized Likelihood Ratio for Exponential Distributions, Generalized Likelihood Ratio for Exponential Distributions with Finite Correction	N/A	10	ARL_0_ set to 50,000 to limit false-positives
**Sequential Changepoint Detection with Nonparametric Methods**	Both	Models: Mann-Whitney, Mood, and Cramer von Mises	N/A	6	ARL_0_ set to 50,000 to limit false-positives
**Batch Changepoint Detection with Parametric Methods**	Both	Models: Student, Bartlett, Generalized Likelihood Ratio	N/A	6	Default alpha level 0.05 used
**Batch Changepoint Detection with Nonparametric Methods**	Both	Models: Mann-Whitney, Mood, Kolmogorov-Smirnov, and Cramer von Mises	N/A	8	Default alpha level 0.05 used
**Bayesian Changepoint Analysis**	Both	Prior of change point probability on the probability of a change point in the sequence (p_0_) = (0, 0.1, 0.2, 0.3, 0.4, 0.5, 0.6, 0.7, 0.8, 0.9, 1.0)	Posterior probability at which changepoint occurs = (0.00, 0.05, 0.10, 0.15, 0.20, 0.25, 0.30, 0.35, 0.40, 0.45, 0.50, 0.55, 0.60, 0.65, 0.70, 0.75, 0.80, 0.85, 0.90, 0.95)	440	

The first general set of algorithms arose from the changepoints package in R [20]. These algorithms test the time series for At Most One Change (AMOC) by examining either a change in the mean of the time series, the variance of the time series, or both. For all three of these sub-methods, the distribution of the data was assumed to be normal. The second set of statistical algorithms were the sequential and batch processing of parametric and non-parametric methods from the cpm package in R [21]. In batch processing, the data is always retrospective and changepoints are calculated from the data as a whole (i.e. all observations in the time series). In sequential processing, the individual data points are received and processed over time until a changepoint is detected (i.e. at each ordered observation, a decision is made whether a change has occurred). The models used in both of these methodologies assume statistical independence between data points in the series, a criteria which may not be strictly met with surface EMG, but was assumed to be true for the current purposes of EMG onset detection. The individual methodologies applied from both batch and sequential processing can be viewed in Table 2. The third set of statistical algorithms were a Bayesian analysis of change points in a time series [22, 23] as implemented in the bcp package in R [23, 24]. As opposed to other methods tested in this study, Bayesian procedures do not produce a point estimate of the EMG onset; instead, a probability distribution is produced and the researcher can select their probability level for onset determination. In this implementation, the posterior means are updated after each partition within the time series. In the present study, the parameter of the prior (hyperparameter γ or *p*_0_) is systematically varied so that the ability to detect a small number of changes (*p*_0_ low) and a large number of changes (*p*_0_ high) are examined [22]. In practice, the parameter can alter the sensitivity and specificity of the algorithm for EMG onset detection. The method also has the capability of accounting for differing levels of signal-to-noise (hyperparameter *w*_0_) which can theoretically be altered based upon the characteristics of the underlying signal. This hyperparameter of the algorithm was set at 0.2 as prescribed by previous work in Bayesian changepoint analysis [22, 25].

Each general algorithm type has a number of parameters which alter how EMG onset is determined and defined. The range of parameters within each algorithm are adapted from the methods typically employed in the EMG literature (in the case of standard algorithms) or are a systematic exploration of the iterative changes available within individual statistical packages (in the case of statistically-oriented algorithms).

### Statistical analysis & algorithm evaluation

Given the large number of EMG onset algorithms examined, an iterative process was used to down-select appropriate algorithms for further analysis. The down-select process was identical for experimental and simulated EMG except for step 3, which was omitted for simulated EMG. The followings steps were used to down-select algorithms: 1) the root mean square error (RMSE) between algorithm-determined EMG onset and visual onset was calculated and algorithms with the highest mean RMSE (top 90% of the 605 algorithm iterations) were removed; 2) algorithms which either detected no EMG onset or detected EMG onset at the first index point more than 25% of the time were removed; 3) for experimental EMG, a linear regression model with algorithm-detected onset and EMG signal-to-noise ratio were used to predict visual onset detection and any algorithms which had statistically significant (p < 0.05) effects for signal-to-noise ratio were removed from further analysis. The signal-to-noise ratio was calculated as the amplitude of the signal during ‘quiet’ EMG in the first 500 ms of data collection in ratio to the maximum amplitude observed during the trial. Despite the uncomplicated nature of the movements performed, the low resistance (2.3 kg) resulted in a reasonable range of signal-to-noise ratios (1.8–78.4). Step 3 was performed with the experimental EMG as the goal of the present investigation was to determine a set of algorithms which produce accurate results independent of signal quality. Any attempt to replicate this regression procedure for the simulated EMG would have resulted in an invalid analysis due to a lack of variance in the dependent variable (e.g. the variance of objective onset in simulated EMG is essentially ‘0’ since all EMG turns ‘on’ at 0.5 seconds).

The remaining algorithms for both experimental and simulated EMG datasets were assessed by determining the mean difference between the algorithm’s determined onset and the ‘gold standard’ visual onset (for experimental data) or objective onset (for simulated data) and the associated parametric 95% confidence intervals. In each case, the accuracy of the algorithm was assessed as the mean difference’s proximity to ‘0’ and the reliability was assessed by examining the width of the 95% confidence intervals (more narrow intervals indicate greater algorithm reliability). All statistical analyses were performed in R [26].

## Results

**Fig 1:**
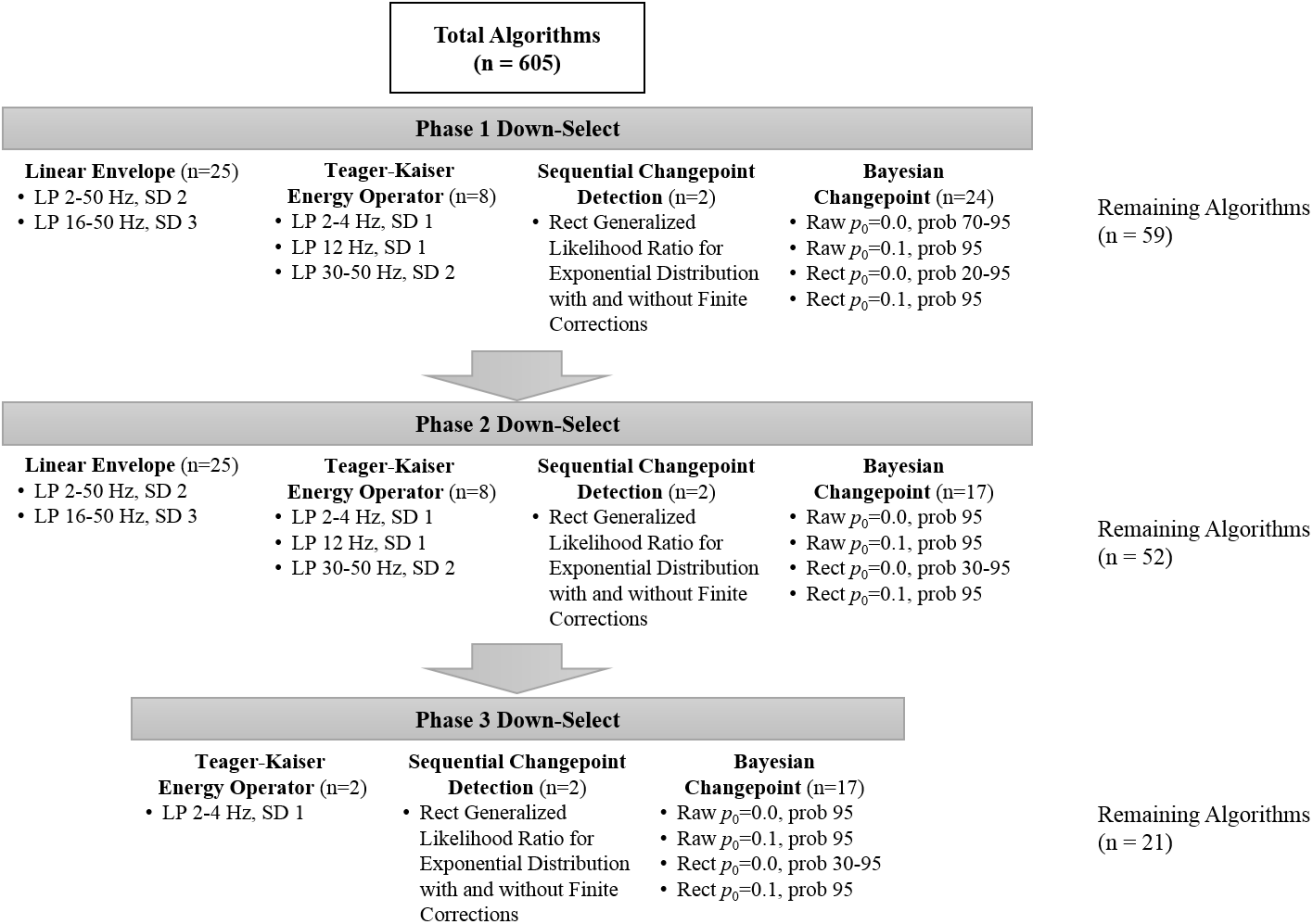
Experimental EMG iterative down-selection process based on root mean square error (RMSE) (Phase 1), clearly aberrant EMG onset detection (Phase 2), and algorithms impacted by the signal-to-noise ratio (Phase 3). Abbreviations: Raw = raw band-pass filtered EMG; Rect = full-wave rectified EMG; p_0_ = parameter of the prior on changepoint probability; Prob = posterior probability for EMG onset; LP = low pass filter frequency; SD = standard deviation of time series for EMG onset.

**Fig 2:**
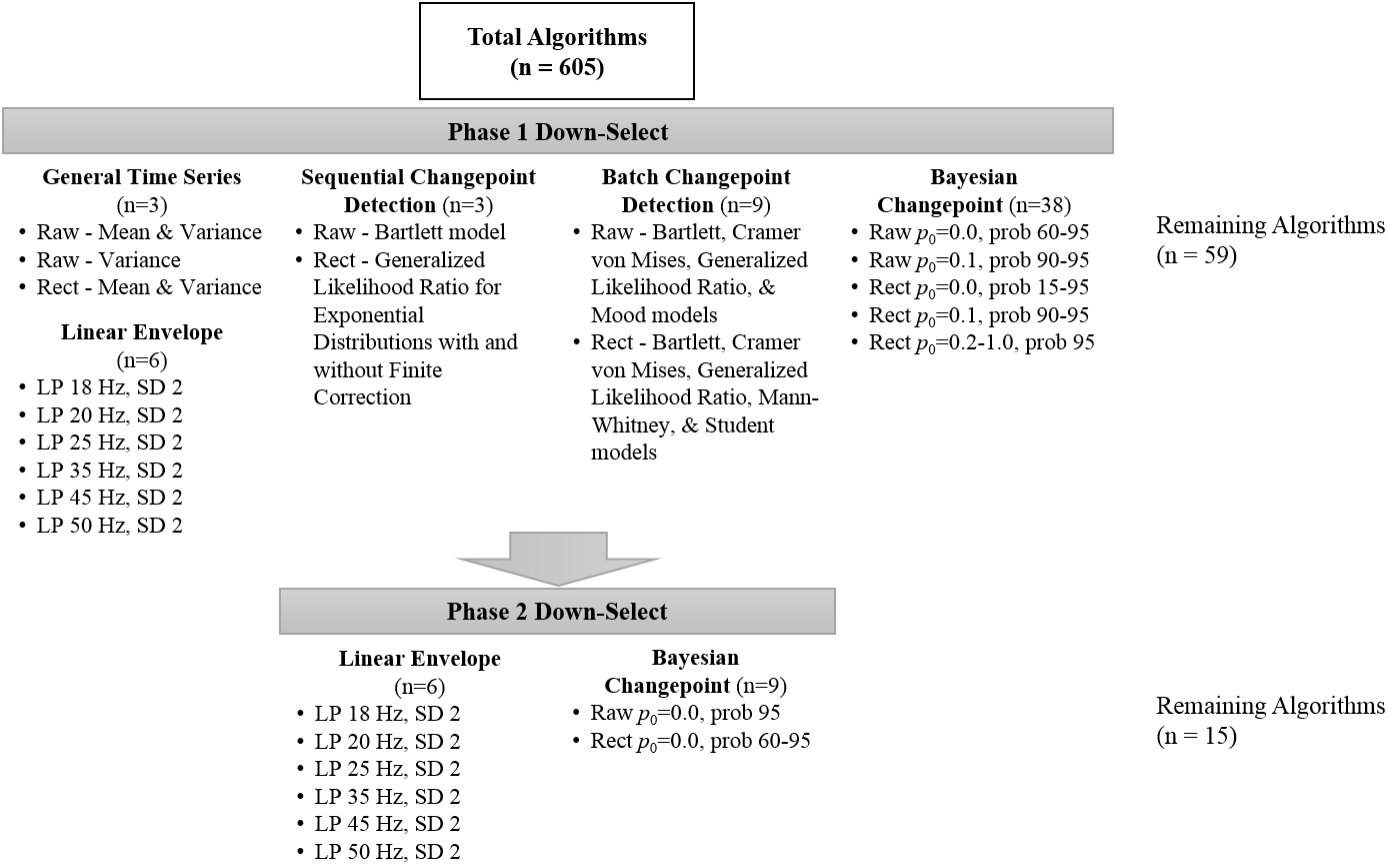
Simulated EMG iterative down-selection process based on root mean square error (RMSE) (Phase 1) and clearly aberrant EMG onset detection (Phase 2). Abbreviations: Raw = raw band-pass filtered EMG; Rect = full-wave rectified EMG; p_0_ = parameter of the prior on changepoint probability; Prob = posterior probability for EMG onset; LP = low pass filter frequency; SD = standard deviation of time series for EMG onset.

The results of the algorithm down-selection process for experimental EMG and simulated EMG can be seen in Fig 1 and Fig 2, respectively.

**Fig 3:**
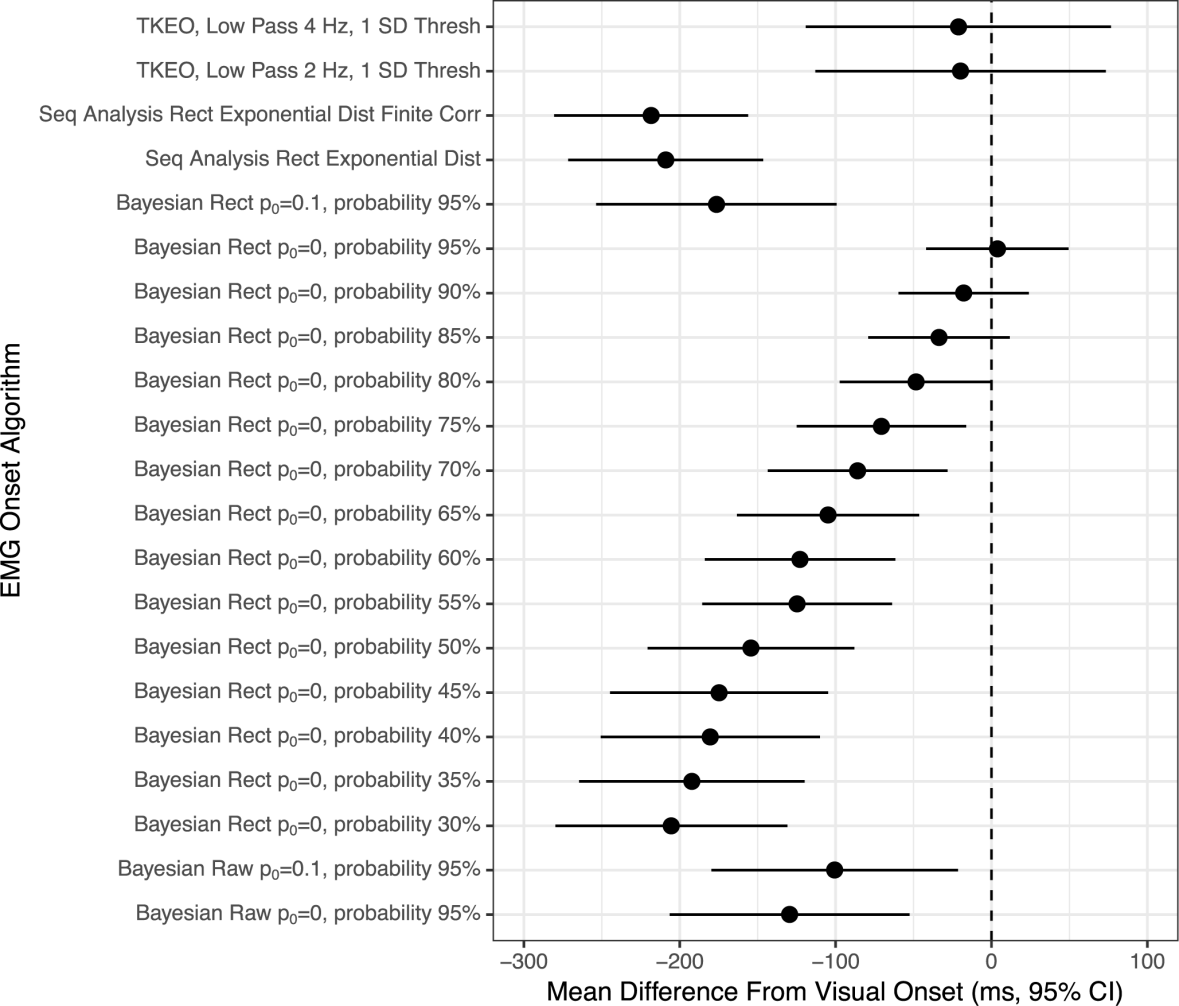
Forest plot of the mean difference between visual EMG onset and algorithm-determined EMG onset for experimentally collected surface EMG. Circle indicates mean difference and bands are the parametric 95% confidence intervals. The dashed line at ‘0’ corresponds to perfect agreement between methodologies. Intervals crossing ‘0’ indicate no statistical difference between methodologies. Interval width corresponds to the reliability of the estimate. Abbreviations: TKEO = Teager-Kaiser energy operator preconditioning, Low Pass = low pass filter frequency, Thresh = threshold for onset determination, Raw = raw EMG, Rect = full-wave rectified EMG, Seq = Sequential analysis of data, Dist = Distribution, Corr = Correction, *p*_0_ = parameter of the prior on changepoint probability.

**Fig 4:**
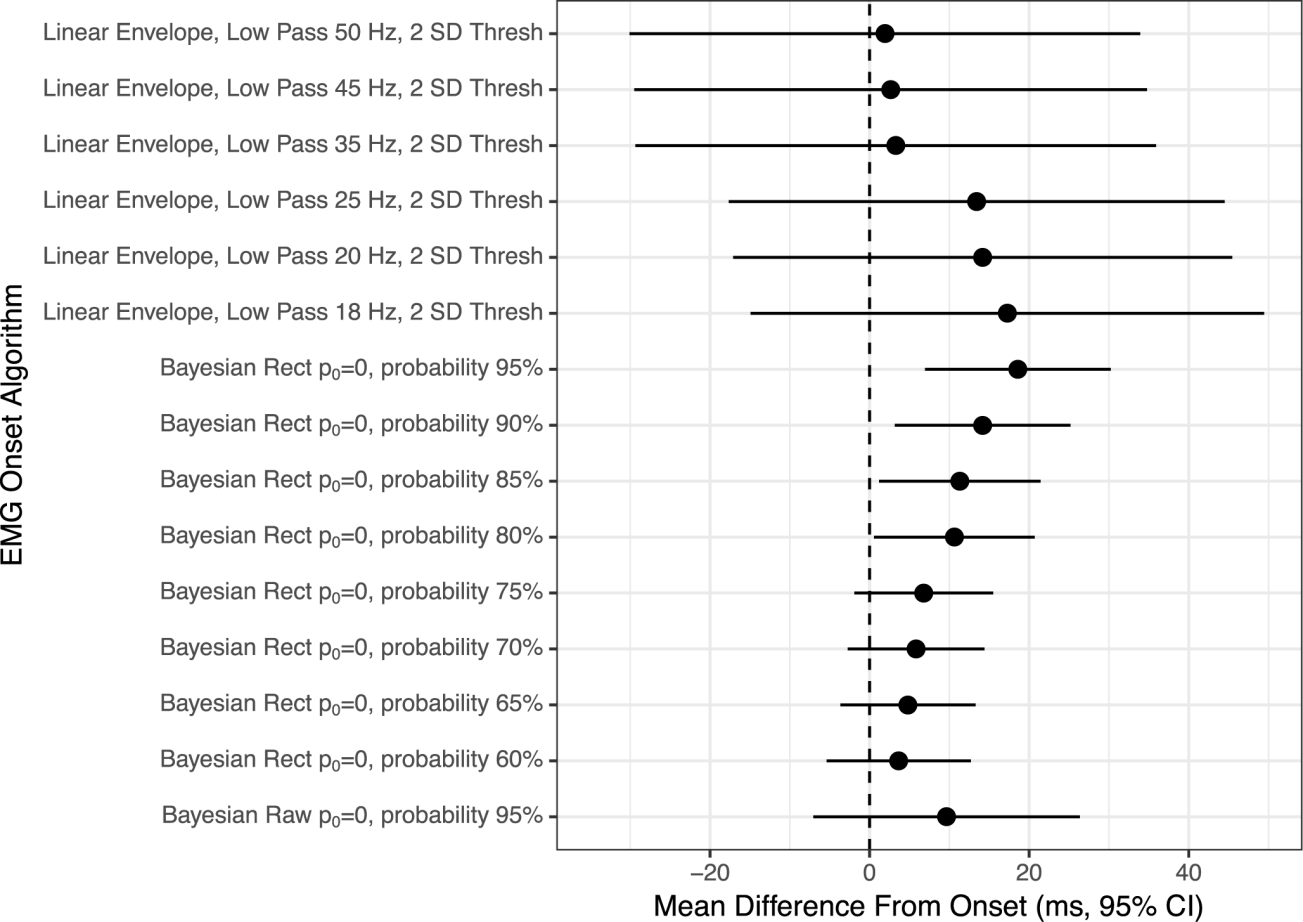
Forest plot of the mean difference between known EMG onset and algorithm determined EMG onset for simulated EMG. Circle indicates mean difference and bands are the parametric 95% confidence intervals. The dashed line at ‘0’ corresponds to perfect agreement between methodologies. Intervals crossing ‘0’ indicate no statistical difference between methodologies. Interval width corresponds to the reliability of the estimate. Abbreviations: Low Pass = zero-lag low pass Butterworth filter, Thresh = threshold for onset determination, Raw = raw EMG, Rect = full-wave rectified EMG, *p*_0_ = parameter of the prior on changepoint probability.

For experimental EMG, the down-selection process rendered 21 algorithm iterations (out of 605) which were determined suitable for more in-depth analyses. The down-select process for simulated EMG produced 15 algorithm iterations (out of 605) which were further assessed. The mean difference from visual onset (± 95% confidence intervals) for the experimental EMG and simulated EMG can be reviewed in Figs 3 and 4, respectively.

For experimental EMG, six of the algorithms were statistically indistinguishable from visual determination, two TKEO algorithms and four Bayesian algorithms (Fig 3). However, the two TKEO algorithms also had the widest confidence intervals contained within the final down-select, suggesting that they have the lowest reliability of the algorithms in Fig 3. When compared with visual determination of EMG onset, the Bayesian Changepoint method (p_0_ = 0.0) used on rectified EMG performed best with the probability of onset threshold set within the 80–95% range.

**Fig 5:**
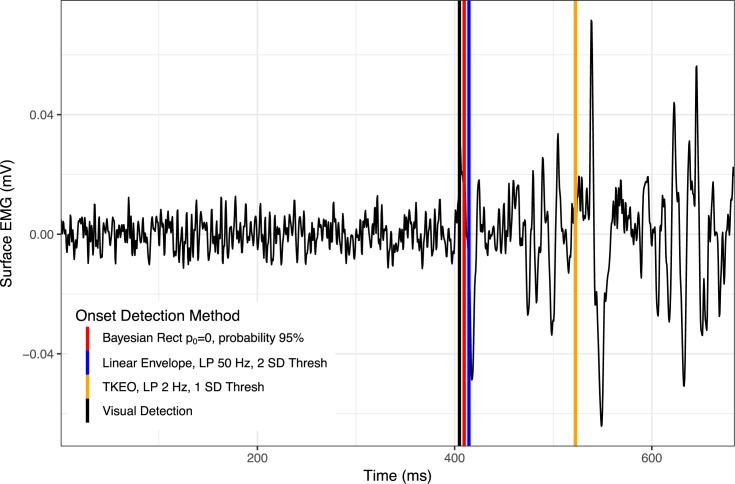
EMG trace in a low noise environment with example onset determinations. Time series length has been substantially cropped, focusing on the time of onset, in order to increase the visibility of onset determination for various methods. Abbreviations: Rect = full-wave rectified EMG; p_0_ = parameter of the prior on changepoint probability; LP = low pass filter frequency; SD = standard deviation of time series for EMG onset; Thresh = threshold for EMG onset.

**Fig 6:**
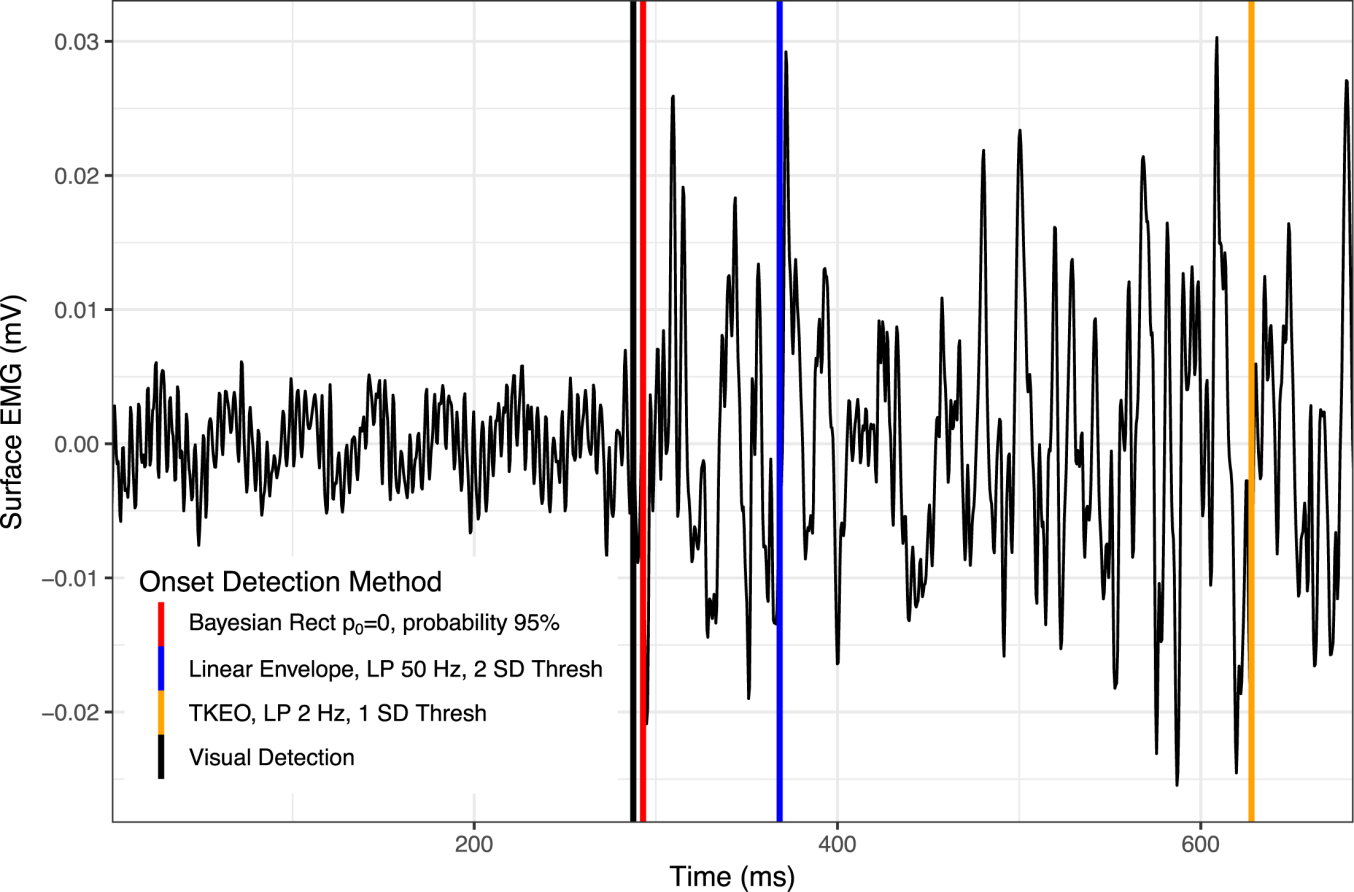
EMG trace in a moderate noise environment with example onset determinations. Time series length has been substantially cropped, focusing on the time of onset, in order to increase the visibility of onset determination for various methods. Abbreviations: Rect = full-wave rectified EMG; p_0_ = parameter of the prior on changepoint probability; LP = low pass filter frequency; SD = standard deviation of time series for EMG onset; Thresh = threshold for EMG onset.

The simulated EMG analysis indicated that only iterations of the linear envelope method and Bayesian changepoint analysis returned even moderately suitable results (Figs 2 and 4). After the final down-select, 11 algorithms were statistically indistinguishable from the objectively known onset of EMG activity. However, all six of the linear envelope algorithms produced exceptionally wide confidence intervals, making them unsuitable for real-world analysis. The algorithm with the most narrow confidence intervals which were also indistinguishable from known EMG onset was the Bayesian changepoint algorithm (p_0_ = 0.0) used on rectified EMG with an onset probability of 65%. All Bayesian changepoint algorithms (p_0_ = 0.0) used on rectified EMG with an onset probability of 60–95% had suitably narrow confidence intervals, indicating high levels of reliability (Fig 4). Example traces of surface EMG with their associated onset determinations for high-performing algorithms are displayed in Figs 5 and 6.

## Discussion

The present study examines various iterations of previously-reported and novel statistical algorithms for EMG onset. The goal was to discover an algorithm or class of algorithms that are accurate, reliable and able to be used in the majority of cases where EMG is collected. While previously reported algorithms produced reasonable results in some cases (Figs 3 and 4), the present analysis suggests that the Bayesian changepoint analytic method produces the most accurate and reliable results after the EMG signal has been full-wave rectified. This result holds true regardless of whether consensus human visual detection is used as the gold standard or a simulated EMG with a known onset is used.

The initial algorithm down-select was coarsely performed by eliminating the top 90% of algorithms with the highest mean RMSE. This step was not intended to determine the best algorithms, but rather to eliminate the algorithms which clearly performed poorly. However, this step alone suggested that the Bayesian changepoint algorithms were a promising analytic technique since it produced 14 and 13 of the top 25 onset algorithms in the experimental and simulated datasets, respectively. This clearly superior performance may be a result of the unique product partition model used within the Bayesian changepoint algorithms to define the different ‘blocks of data’. An imperfect analogy for this product partition model to standard EMG onset analyses would be an automated sliding window function which iterates until an ‘ideal’ window length and overlap is determined for a particular time series. The secondary down-select, determined *a priori*, was the elimination of algorithms that detected no muscle activity or muscle activity at the first time point in the data-series, which is clearly aberrant. The third down-select, performed only in experimental data, was driven by theory and the goal of the present manuscript which was to determine algorithms which are valid irrespective of the quality of surface EMG signal or collection methodology. Thus, multiple linear regression was used with algorithm-determined EMG onset and the signal-to-noise ratio of the EMG time series as co-variates to predict the gold standard for EMG onset detection (i.e. visual detection). If the regression analysis indicated that the signal-to-noise variable significantly impacted the analysis (i.e. p < 0.05), that analytic technique was removed from further analysis. Using null-hypothesis testing to eliminate an algorithm based on regression coefficients is a coarse measure. For example, a more subjective but informative method may have been to plot RMSE against the signal-to-noise data in either a histogram or scatter plot format. This type of visual analysis is more informative as it will indicate which methodologies are highly effective under various levels of signal noise. Methodologies in this vein were not performed as they are inherently subjective, more time consuming, and the interest of the present analysis was to find algorithms that performed well regardless of signal quality.

It is remarkable that both the experimental dataset and the simulated dataset suggest that the same Bayesian changepoint algorithms with p_0_ = 0.0 and onset probability threshold ranging from 60–95% produce highly reliable and generally accurate results when compared to more standard approaches. It is the opinion of the authors that while accuracy and reliability of an EMG onset algorithm are both important, it is generally more acceptable to have a slightly biased algorithm which is highly reliable compared to an algorithm which is, on average, more accurate but has wider confidence intervals. With that bias in mind, we believe that the results in Figs 3 and 4 indicate that Bayesian changepoint analysis is a superior class of EMG onset algorithms when comparing a singular change in the time series.

### Bayesian changepoint analysis

The Bayesian changepoint analysis implemented in the current study is based on the original work by Barry and Hartigan [22] and extended as the R package bcp [23]. These analytical methods have been applied to a wide variety of fields, from genomics [24] to climate change [27] and investigations of the National Hockey League demographics [28]. In general, the analysis provides a posterior probability for the presence of a changepoint within a given partition of the time series. The posterior probability is updated after each partition is iterated and are conditional on the current partition. Two hyperparameters within Bayesian changepoint analysis can be ‘tuned’ to increase their efficacy for a given situation: *p*_0_ and *w*_0_. The exact derivation and use of these hyperparameters is detailed in the original work by Barry and Hartigan [22]; in the context of EMG onset, higher *p*_0_ values are effective when there are many changepoints to detect and higher *w*_0_ values are effective when the signal-to-noise ratio of the underlying EMG signal is low [22, 23]. Both *p*_0_ and *w*_0_ are bounded from 0–1.

In the confines of the present study, *w*_0_ was not systematically altered and the default 0.2 level was used as prescribed by previous work [22, 25]. The *p*_0_ hyperparameter was iteratively changed from 0–1 (Table 2). Given the current study which only examined a singular “EMG onset” or changepoint in a time series, it should be unsurprising that *p*_0_ = 0.0 rendered the most reliable and accurate results. The present study also iteratively examined the most appropriate cut-point for the posterior probability, with various probabilities being more reliable in different data sets (experimental vs. simulated, see Figs 3 and 4). In practice, the selection of an appropriate posterior probability set-point may also be guided by the underlying theory and belief of the researcher regarding the desired level of confidence in the data.

The clearly superior performance of Bayesian changepoint analysis for the determination of surface EMG onset in both experimental and simulated data indicates that this methodology deserves greater study. Research which examines multiple muscle onsets and offsets in dynamic tasks should provide evidence that the *p*_0_ hyperparameter needs to be varied depending on the type of EMG data collected. The *w*_0_ hyperparameter may be able to be altered depending upon signal quality or the signal-to-noise ratio of the underlying EMG data. The differential fixation of these two hyperparameters will also impact the overall algorithm accuracy and reliability. In theory, a function could be derived which modifies the hyperparameters based upon the underlying characteristics of the EMG signal being evaluated. Future research should explore this possibility as it may allow for the most reliable and flexible analysis of EMG onset across laboratories and in clinical applications.

### Future work

Many of the novel algorithms tested in the current manuscript arise from areas of econometrics, genomics and statistical changepoint analysis. The superior performance of the Bayesian changepoint algorithms reinforce the notion that biomechanics and motor control researchers should continually assess the state-of-the art in adjacent or seemingly unrelated fields to leverage the computational or experimental advances in those fields. Computational work in the areas of non-linear dynamics [29, 30], recursive neural networks [31, 32] and network dynamics [33] may be especially fruitful for extracting EMG onset timing.

## Conclusions

The current study examined a large number of existing and statistical algorithm iterations for use in determining surface EMG onset. While previously proposed linear envelope and TKEO methods generally performed well, the Bayesian changepoint class of algorithms showed the most promise. When only one muscle onset needs to be detected in a time series, as may be the case in analyzing discrete movements (e.g. a drop-landing task, reaction time detection, etc.) the Bayesian changepoint algorithm with hyperparameters *p*_0_ = 0 and *w*_0_ = 0.2 performed best at the 60–90% confidence level. Future research needs to explore iterations of Bayesian changepoint analysis in more complicated EMG waveforms with both EMG ‘onsets’ and ‘offsets’ to determine its viability in more complex tasks.

## References

[1] Sutherland DH. The evolution of clinical gait analysis part l: kinesiological EMG. Gait Posture. 2001;14(1):61–70. 11378426 10.1016/s0966-6362(01)00100-x

[2] Lyons K, Perry J, Gronley JK, Barnes L, Antonelli D. Timing and relative intensity of hip extensor and abductor muscle action during level and stair ambulation An EMG study. Physical therapy. 1983;63(10):1597–605. 6622534 10.1093/ptj/63.10.1597

[3] Bennell K, Duncan M, Cowan S, McConnell J, Hodges P, Crossley K. Effects of vastus medialis oblique retraining versus general quadriceps strengthening on vasti onset. Medicine and science in sports and exercise. 2010;42(5):856–64. doi: 10.1249/MSS.0b013e3181c12771 19997004 10.1249/MSS.0b013e3181c12771

[4] Winter DA. Biomechanics and motor control of human movement: John Wiley & Sons; 2005.

[5] Solnik S, Rider P, Steinweg K, DeVita P, Hortobágyi T. Teager–Kaiser energy operator signal conditioning improves EMG onset detection. Eur J Appl Physiol. 2010;110(3):489–98. doi: 10.1007/s00421-010-1521-8 20526612 10.1007/s00421-010-1521-8 PMC2945630

[6] Li X, Zhou P, Aruin AS. Teager–Kaiser energy operation of surface EMG improves muscle activity onset detection. Annals of biomedical engineering. 2007;35(9):1532–8. doi: 10.1007/s10439-007-9320-z 17473984 10.1007/s10439-007-9320-z

[7] Zhang X, Zhou P. Sample entropy analysis of surface EMG for improved muscle activity onset detection against spurious background spikes. J Electromyogr Kinesiol. 2012;22(6):901–7. doi: 10.1016/j.jelekin.2012.06.005 22800657 10.1016/j.jelekin.2012.06.005 PMC3514830

[8] Kaiser JF, editor On a simple algorithm to calculate the `energy' of a signal. International Conference on Acoustics, Speech, and Signal Processing; 1990 Apr 3–6; Albuquerque, New Mexico (USA)1990.

[9] Kaiser JF, editor Some useful properties of Teager's energy operators. International Conference on Acoustics, Speech, and Signal Processing; 1993 April 27–30; Vancouver, BC (Canada)1993.

[10] Pincus SM, Gladstone IM, Ehrenkranz RA. A regularity statistic for medical data analysis. J Clin Monitor. 1991;7(4):335–45. 10.1007/BF01619355 1744678

[11] Richman JS, Moorman JR. Physiological time-series analysis using approximate entropy and sample entropy. Am J Physiol Heart Circ Physiol. 2000;278(6):H2039–49. 10843903 10.1152/ajpheart.2000.278.6.H2039

[12] Boxtel G, Geraats L, Berg‐Lenssen M, Brunia C. Detection of EMG onset in ERP research. Psychophysiol. 1993;30(4):405–12. 10.1111/j.1469-8986.1993.tb02062.x 8327626

[13] Hodges PW, Bui BH. A comparison of computer-based methods for the determination of onset of muscle contraction using electromyography. Electroencephalogr Clin Neurophysiol. 1996;101(6):511–9. 9020824 10.1016/s0013-4694(96)95190-5

[14] Box GE. Robustness in the strategy of scientific model building. Robustness in statistics. 1979;1:201–36.

[15] Almog M, Korngreen A. Is realistic neuronal modeling realistic? J Neurophysiol. 2016;116(5):2180–209. doi: 10.1152/jn.00360.2016 27535372 10.1152/jn.00360.2016 PMC5102320

[16] Neto OP, Christou EA. Rectification of the EMG signal impairs the identification of oscillatory input to the muscle. J Neurophysiol. 2010;103(2):1093–103. PubMed Central PMCID: PMCPMC2822682. doi: 10.1152/jn.00792.2009 20032241 10.1152/jn.00792.2009 PMC2822682

[17] Halliday DM, Farmer SF. On the need for rectification of surface EMG. J Neurophysiol. 2010;103(6):3547; author reply 8–9. doi: 10.1152/jn.00222.2010 20530508 10.1152/jn.00222.2010

[18] Boonstra TW. The nature of periodic input to the muscles. J Neurophysiol. 2010;104(1):576-. doi: 10.1152/jn.00258.2010 20610794 10.1152/jn.00489.2010 PMC2904218

[19] Dakin CJ, Dalton BH, Luu BL, Blouin J-S. Rectification is required to extract oscillatory envelope modulation from surface electromyographic signals. J Neurophysiol. 2014;112(7):1685–91. doi: 10.1152/jn.00296.2014 24990563 10.1152/jn.00296.2014

[20] Killick R, Eckley I. changepoint: An R package for changepoint analysis. J Stat Softw. 2014;58(3):1–19.

[21] Ross GJ. Parametric and nonparametric sequential change detection in R: The cpm package. J Stat Softw. 2015;66(3):1–20.

[22] Barry D, Hartigan JA. A Bayesian analysis for change point problems. J Am Statist Assoc. 1993;88(421):309–19.

[23] Erdman C, Emerson JW. bcp: an R package for performing a Bayesian analysis of change point problems. J Stat Softw. 2007;23(3):1–13.

[24] Erdman C, Emerson JW. A fast Bayesian change point analysis for the segmentation of microarray data. Bioinformatics. 2008;24(19):2143–8. doi: 10.1093/bioinformatics/btn404 18667443 10.1093/bioinformatics/btn404

[25] Yao Y-C. Estimation of a noisy discrete-time step function: Bayes and empirical Bayes approaches. Ann Stat. 1984:1434–47.

[26] R Core Team. R: A language and environment for statistical computing. Vienna, Austria: R Foundation for Statistical Computing; 2015.

[27] Kortsch S, Primicerio R, Beuchel F, Renaud PE, Rodrigues J, Lønne OJ, et al Climate-driven regime shifts in Arctic marine benthos. PNAS. 2012;109(35):14052–7. doi: 10.1073/pnas.1207509109 22891319 10.1073/pnas.1207509109 PMC3435174

[28] Addona V, Yates PA. A closer look at the relative age effect in the National Hockey League. J Quant Anal Sports. 2010;6(4):1–17.

[29] Donges JF, Heitzig J, Beronov B, Wiedermann M, Runge J, Feng QY, et al Unified functional network and nonlinear time series analysis for complex systems science: The pyunicorn package. Chaos. 2015;25(11):113101 doi: 10.1063/1.4934554 26627561 10.1063/1.4934554

[30] Gao Z-K, Cai Q, Yang Y-X, Dang W-D, Zhang S-S. Multiscale limited penetrable horizontal visibility graph for analyzing nonlinear time series. Sci Rep. 2016;6:35622 doi: 10.1038/srep35622 27759088 10.1038/srep35622 PMC5069474

[31] Funahashi K-i, Nakamura Y. Approximation of dynamical systems by continuous time recurrent neural networks. Neural Net. 1993;6(6):801–6.

[32] Socher R, Lin CC, Manning C, Ng AY, editors. Parsing natural scenes and natural language with recursive neural networks. Proceedings of the 28th international conference on machine learning (ICML-11); 2011.

[33] Gao Z-K, Small M, Kurths J. Complex network analysis of time series. Europhys Lett. 2016;116(5):50001.

